# Pay for performance: will dentistry follow?

**DOI:** 10.1186/1472-6831-10-9

**Published:** 2010-04-28

**Authors:** Andreea Voinea-Griffin, Jeffrey L Fellows, Donald B Rindal, Andrei Barasch, Gregg H Gilbert, Monika M Safford

**Affiliations:** 1 University of Alabama at Birmingham, Department of General Dental Sciences, Birmingham, USA; 2 Kaiser Permanente Center for Health Research, Portland, USA; 3 Health Partners Research Foundation, Minneapolis, USA; 4 University of Alabama at Birmingham, School of Medicine, Birmingham, USA

## Abstract

**Background:**

"Pay for performance" is an incentive system that has been gaining acceptance in medicine and is currently being considered for implementation in dentistry. However, it remains unclear whether pay for performance can effect significant and lasting changes in provider behavior and quality of care. Provider acceptance will likely increase if pay for performance programs reward true quality. Therefore, we adopted a quality-oriented approach in reviewing those factors which could influence whether it will be embraced by the dental profession.

**Discussion:**

The factors contributing to the adoption of value-based purchasing were categorized according to the Donabedian quality of care framework. We identified the dental insurance market, the dental profession position, the organization of dental practice, and the dental patient involvement as structural factors influencing the way dental care is practiced and paid for. After considering variations in dental care and the early stage of development for evidence-based dentistry, the scarcity of outcome indicators, lack of clinical markers, inconsistent use of diagnostic codes and scarcity of electronic dental records, we concluded that, for pay for performance programs to be successfully implemented in dentistry, the dental profession and health services researchers should: 1) expand the knowledge base; 2) increase considerably evidence-based clinical guidelines; and 3) create evidence-based performance measures tied to existing clinical practice guidelines.

**Summary:**

In this paper, we explored factors that would influence the adoption of value-based purchasing programs in dentistry. Although none of these factors were essential deterrents for the implementation of pay for performance programs in medicine, the aggregate seems to indicate that significant changes are needed before this type of program could be considered a realistic option in dentistry.

## Background

Rising costs [1] and variable quality [2] are two of the major challenges faced today by the United States (US) healthcare system. Increasing expenditures without concomitant improvements in public health [3] convinced stakeholders to request more transparency and accountability for their healthcare dollars [4-6]. "Pay-for-performance" (P4P) is a group of value-based purchasing programs that attempt to link provider reimbursement to improvements in healthcare quality. A widely used concept of healthcare that underlies many P4P programs defines quality as "doing the right thing, at the right time, in the right way delivered to the right patient [7]".

In recent years, the concept of paying providers or provider groups for achieving better marks on quality indicators has become common [8,9]. Incentive schemes are now routinely used by managed care organizations (MCOs) and public programs in the US. In a recent study, Rosenthal et al. [10] found that 126 of 225 MCOs, representing more than 80% of managed care enrollees, use P4P programs in their provider contracts [10]. In 2007, the Institute of Medicine (IOM) reported that there were over 100 reward and incentive programs within Medicare in the US [11]. Although still largely experimental, the number of initiatives is growing.

P4P programs have been widely adopted despite the fact that they are based on several unproven assumptions. One is that providers' clinical behavior can actually be changed using financial incentives. Some evidence suggests payment method affects provider behavior [12,13], but these reports may have limited generalizability and the impact on quality has not been reported [14-16]. Several systematic reviews concluded that more research is needed in the area of provider payments. Gosden et al [15] found that compared to capitation, fee-for-service was associated with providing more primary care services, but the impact on the quantity of specialty services was mixed. Moreover, fee-for-service was associated with lower patient satisfaction with access to a physician compared with salary payment. Other studies found no evidence that performance payments were associated with improved primary health care [16,17].

A second assumption is that a link exists between P4P indicators and true improvements in quality. Rosenthal and Frank found little evidence of the effectiveness of paying for quality in healthcare [18]. For example, assessing risk factor levels, a process indicator, is not associated with reducing risk factor levels. Mangione et al [19] found that disease management strategies were associated with better processes of diabetes care but not with improved intermediate outcomes. Only by ameliorating the actual risk factors can health be impacted. This issue is further complicated by the impact on clinical outcomes of numerous patient factors that are not incorporated into quality of care indicators. Moreover, P4P may lead to "gaming" by providers, who may recruit the types of patients who tend to have better outcomes and be compensated for their "performance" rather than providing better quality of care. Provider manipulation has been a challenge confronting the British experiment with P4P [17,20].

Lastly, P4P systems assume that payers can accurately determine which components of care could be positively and negatively impacted by financial incentives and design a payment program whose benefits will outweigh any unintended consequences [21]. But, despite its appeal as a payment method, P4P has proven extremely complex, hard to devise and difficult to implement [22,23]. In addition, there are conflicting results about P4P impact on quality [24-26], ethical concerns [27,28], and mixed provider acceptance [29]. Despite these problems, P4P continues to expand.

Although initially designed for medical care, it is possible that P4P will also be considered for dentistry. The American Dental Association (ADA) has already issued a statement on the prerequisites for acceptance of a P4P program by the profession and closely monitors all related national legislative proposals [30]. History shows that the dental profession often follows in the footsteps of medicine. For example, electronic records and evidence-based care are gaining acceptance by the dental profession after becoming routine in medicine. However, differences in the delivery and payment for medical and dental services must be considered before implementing P4P in dentistry.

## Discussion

A growing body of literature describes value purchasing programs and their implementation in various healthcare systems [9,31,32]. Although provider incentives differ by healthcare system and P4P program, significant design and implementation obstacles exist in every country where P4P was implemented [33,34]. Despite the focus of this analysis on the American system of dentistry, the factors influencing the P4P adoption exist in various degrees in other dental healthcare systems as well.

Providing evidence that P4P improves dental care quality, limits costs, or both, would strengthen the case for its introduction. However, no such evidence existed prior to implementation of P4P in medicine. Since the adoption of value-based purchasing depends on provider involvement, this analysis presents possible challenges of implementing P4P in dentistry from a quality-of-care perspective. One might expect greater provider acceptance if P4P programs are proven to actually reward quality. Thus, we will focus our discussion on the quality aspect of performance-based programs and examine the factors which could influence whether P4P is adopted in dentistry. However, to aid in explaining factors that need to be addressed before P4P is adopted in dentistry, we categorize these factors according to the classic quality of care framework by Donabedian [35], in which quality is evaluated using structure, process and outcomes parameters.

### 1. Structural factors

#### The Insurers

The structure of dental insurance is a major contributing factor in the implementation on any value-based plan. For example, in the United Kingdom (UK), dental care is largely covered by the National Health Services (NHS). Consequently, payment policy changes involving P4P may have a greater influence on UK dental providers than programs in other healthcare systems. In the US, public insurance for dental services is extremely limited; thus, any payment policy initiatives by public payers will have limited impacts on mainstream private dental practice. Moreover, an estimated 44% of adults do not have any dental coverage [36]. The high percentage of out-of-pocket payments in dental practices and the multitude of different insurers per practice diminish the insurers' negotiation power and make the acceptance of performance-based payment programs by dental providers more difficult. Thus, the adoption of P4P in dentistry will be a difficult public policy issue because it would largely have to be implemented by the private sector. This could significantly change if US healthcare reform mandates a certain level of dental services or if the dental insurance market becomes more concentrated.

Variations in service coverage among dental insurance plans complicates matters. This can create a dissonance between the realities of the dental reimbursement system and the very few clinical practice guidelines that do exist. For instance, some plans do not cover dental sealants. The current procedure-based payment for dental services is based on delivery of procedures, which may reward over-treatment of covered procedures. This financial aspect of dental care delivery may be one of the biggest reasons for the delay in P4P in dentistry.

#### The Profession

Both dentists in the community and dental organizations may be substantially affected by the implementation of any P4P program. Consequently, professional organizations have a direct interest in P4P initiatives. Loss of control, cookbook care, inadequate quality assessment standards, and finance-driven practice are common professional concerns. To address them, in 2006 the ADA released a position statement on value-based purchasing entitled "Principles for Pay for Performance or Other Third-Party Financial Incentive Programs [30]". The ADA represents about 69% of US dentists [37]. Ten principles laid out in this document reflect the profession's desire to preserve decisional autonomy and payer's non-interference in the dentist-patient relationship. The document emphasizes dentists' interest in having quality as the goal of any financial-incentive program, in maintaining patients' access to quality care, and in allowing all P4P participation to be voluntary. Moreover, the document expressed a goal that quality indicators should be minimum in number, standard, accepted, clear, measurable, and able to factor in patient risk and compliance. The ADA does not currently provide quality indicators on its website, but does list these components of care: 1) infection control; 2) toxic exposure control; 3) medical emergency procedures; 4) access; 5) privacy; 6) safety; and 7) patient record and documentation. These parameters are merely descriptors of care and not intended for policy development [38]. In addition, ADA House resolution 24 states that any P4P program in dentistry will be challenged due to lack of "generally accepted/universal, credible quality guidelines developed by the profession" [30].

Specialist dental associations have not taken a public position on value-based purchasing, possibly due to the fact that a smaller share of specialists' revenue comes from insurance sources [36], and because the current focus of insurers is on primary care rather than specialty care. An exception is the American Association of Oral and Maxillofacial Surgeons, whose members perform Medicare-reimbursed procedures and will likely be the first dental specialty affected by P4P. The Association is interested in securing fair reimbursement for its members and emphasizes the need for "complete and accurate measurement of the Oral Maxillofacial Surgeons' work value" [39].

#### The Dental Practice

Differences in how medical and dental providers are organized play a significant role in the adoption of quality improvement programs and the potential for P4P implementation. Sixty-five percent of US dental practices are owned and operated by solo practitioners [40,41] as opposed to only about 24% in the medical field [39]. Few MCOs include a dental plan, and large provider groups are less common in dentistry than in medicine [36]. Provider interaction in large physician groups is instrumental to quality improvement [42], but is rarely seen in dentistry [43]; as a result, dental quality initiatives are limited in number and scope and not widely reported in the literature [44-47]. Moreover, quality initiatives in medicine are guideline-based, while in dentistry, peer review is preferred [48], and guidelines are relatively few in number. The lack of information about quality programs and the predominance of solo practice may significantly inhibit the adoption of P4P in dentistry.

#### The Dental Patient

Equally affected but apparently the least involved party in P4P dental programs are the dental patients themselves. The urgency for quality improvement which drove the implementation of P4P in medicine is not currently present in dentistry. Despite limited insurance coverage and wide-spread oral disease, out-of-pocket expenses for dental services are relatively low compared to medical care costs and expected by consumers. This apparent imbalance of the cost-to-value relationship for most people will not be addressed by any P4P system. Thus, it is unlikely that the consumers will organize and demand increased scrutiny on dental services as they have done for medical care.

### 2. Process of care factors

#### Variation in dental care

There is considerable variation in dental treatment modalities, and this variation is difficult to attribute to the types of patients cared for [49,50]. For example, the use of sealants varies greatly among dentists, although overwhelming evidence about their effectiveness exists [51,52]. There is also wide variation in the way early caries are treated [53], despite research proving that remineralization of enamel caries is possible and recommended [51,54]. Two-stage therapy, pre- and post-puberty, for severe Class II malocclusion (prominent front teeth) is still preferred by many orthodontists, despite evidence that the one-stage treatment is shorter, less costly and has the same results [55]. In response to such observations, Bader and Shugars [53,56] suggested that comprehensive approaches to improving consistency across the dental profession would improve quality more than traditional methods, which have typically focused on outlier dentists.

Another possible explanation for the observed differences in care is the inadequate dissemination of recent scientific evidence. Although this might be the case for some dentists, the marginal improvements obtained through provider interventions based solely on education [57] suggest that additional reasons for observed differences exist. Clinical inertia, defined as "recognition of a problem but not acting to treat or prevent the problem in the desired manner based on current evidence" [58], may contribute to delays in the large-scale implementation of recommended care and has been offered as an alternative explanation for observed variations. A notable example is the use of dental sealants, estimated to prevent 80% of pit and fissure caries and promoted by the ADA since 1976. After 30 years, countless educational programs and mounting evidence, the dental profession has achieved a mere 50% of children receiving this service [59]. Similarly, third molar prophylactic extractions continue to be recommended despite evidence against this treatment [60].

Other compelling explanations for the existing variation in care exist. Studies have shown that the distribution of dental services is different when payment methodology changes [61,62]. In addition, the variation in treatment decisions has significant cost implications [50], which seem to be due at least in part to the paucity of evidence regarding many common dental treatments [63].

#### Evidence-Based Dentistry

Implementing a P4P program has been shown to reduce variation in medical care [64,65]. Clinical guidelines, defined by the IOM as "systematically developed statements to assist practitioner and patient decisions about appropriate health care for specific clinical circumstances" [66], may reduce the variation in the delivery of care. In general, guidelines are developed based on evidence or expert opinion, absent sufficient evidence, According to the ADA, evidence-based dentistry is "an approach to oral health care that requires the judicious integration of systematic assessments of clinically relevant scientific evidence, relating to the patient's oral and medical condition and history, with the dentist's clinical expertise and the patient's treatment needs and preferences" [67].

Dentistry has an abundance of published research but in most areas lacks the strong evidence that comes from randomized controlled trials. The ADA has begun to advocate for the practice of evidence-based dentistry, and to educate dentists about best practices [67,68]. Yet, the ADA lags behind other organizations in providing dentists with clinical guidance (Table 1). On its web page, the ADA has links to 89 topics, each with several literature reviews, but none endorsed by the organization. Within the posted reviews, many reports cite insufficient evidence. For example, single crown restorations were reported to be the most frequent dental procedure after prophylaxis and periodic oral evaluation [69]. Of the six reviews addressing various aspects of single crown restorations in permanent teeth, four report insufficient evidence, whereas the other two report only fair evidence. The ADA makes evidence-based clinical recommendations in only three areas: (1) prevention of infective endocarditis, (2) use of sealants and (3) professionally-applied topical fluoride.

**Table 1:** Stomatognathic Clinical Guidelines in the National Guideline Clearinghouse™[81]

Clinical guidelines directly related to the practice of dentistry	**No**.
American Dental Association	2
American Academy of Pediatric Dentistry	22
American Cleft Palate Craniofacial Association	1
American Academy of Pediatrics	1
American Academy of Sleep Apnea	1
Center for Disease Control	1
US Preventive Services Task Force	2
New York State Department of Health	2
Health Partners	4
University of Texas at Austin	1
Non US Government Agencies	8

**Areas covered by other stomatognathic clinical guidelines**	

Preventive Services	12
Cancer	3
Infectious Diseases	7

**TOTAL**	**67**

The dearth of evidence-based recommendations for dental care can also be illustrated by the following examples. The Agency for Healthcare Research and Quality (AHRQ) develops and posts evidence-based practice reports on its webpage. Of the 159 reports, only 1 is dentistry-related and reports insufficient evidence to strongly recommend its implementation. The US Preventive Services Task Force is an independent panel of experts in primary care and prevention sponsored by the AHRQ to review the evidence. This group rates the strength of that evidence, and submits its recommendations. Of the posted 103 recommendations, 2 are dentistry-related, of which only one was rated to have sufficient evidence to recommend the practice [70] (Table 2). Similarly, the Cochrane Collaborative Group reviewed 82 topics on oral health [71] and found insufficient evidence on many of them. Under this circumstance, identifying best evidence and incorporating it in the daily clinical practice is challenging and it is not systematically done by many dentists [72].

**Table 2:** Dental care evidence reports and recommendations [70,82]

AHRQ Evidence report	Level of Evidence
Effectiveness of Antimicrobial Adjuncts to Scaling and Root Planning Therapy for Periodontitis	Insufficient

**USPSTF Recommendations**	**Level of Evidence**

The USPSTF recommends that primary care clinicians prescribe oral fluoride supplementation at currently recommended doses to preschool children older than 6 months of age whose primary water source is deficient in fluoride.	Fair
The USPSTF concludes that the evidence is insufficient to recommend for or against routine risk assessment of preschool children by primary care clinicians for the prevention of dental disease.	Insufficient

A change in culture regarding quality improvement in the medical profession started, although it has not yet been embraced by all physicians [73]. A similar change will be necessary before any P4P programs could be realistically considered in dentistry.

### 3. Outcome factors

#### Outcome indicators

Quality assessment is a prerequisite for quality improvement. Compared with outcomes, structure and processes are easier to measure and most quality improvement initiatives rely heavily on this type of indicator. Yet, high quality structure and processes do not necessarily result in high quality outcomes. Outcome indicators have been the most difficult to develop and measure [74]. A study on the development of effectiveness of care using dental plan data resulted in 7 measures, 3 of which were outcome measures [75]. Despite reasonably reliability and sensitivity, the large scale adoption of these measures to evaluate providers' performance was hindered by reliance on and limitation of diagnostic information in the administrative datasets.

Currently there are very few clinical outcomes indicators in dentistry. Among them are the Decayed, Missing, Filled Surfaces (DMFS), Periodontal Index, Gingival Bleeding Index, and Oral Hygiene Index. Of the 352 outcomes indicators publicly available on the National Quality Measures Clearinghouse, only 9 are related to oral and dental diseases and 3 are clinical outcome indicators relevant to the practice of dentistry [76] (Table 3).

**Table 3:** Clinical outcome indicators for pediatric restorative dentistry [79]

Indicator
Percentage of deciduous teeth extracted (for pathological reasons) within 6 months following pulpotomy treatment, during the time period under study.
Percentage of teeth requiring re-treatment (restoration, endodontic or extraction, but not including Pit & Fissure Sealants) within 24 months of the initial fissure sealant treatment.
Percentage of teeth requiring repeat fissure sealant treatment within 24 months of the initial fissure sealant treatment.

#### Clinical markers

One factor which makes the development of outcomes indicators particularly challenging in dentistry is the prevalence of chronic dental conditions, which do not have established severity markers as do chronic medical conditions like diabetes or hypertension. For diabetes management, the hemoglobin A_1c _level is an established indicator of disease control. Blood pressure is used for hypertension control. Such indicators do not exist for managing dental caries, which is the most prevalent disease in dentistry. Caries progress slowly and they are typically treated by several dentists over decades. Moreover, most dentists do not currently record clinical information needed for outcomes assessment, such as caries depth, caries activity, change in caries rate, caries risk assessment, patient symptoms and past treatments including preventive treatments. Other quality indicators such as biological and psychosocial outcomes are rarely recorded [77]. Thus, it is difficult to create and validate meaningful outcome indicators and enhance the level of evidence in the treatment of caries and other chronic dental conditions.

#### Diagnostic codes

Procedure codes developed by the ADA [78] are widely accepted by third-party payers for reimbursement for dental services in the US. However, unlike medicine, no standardized diagnostic codes exist for dentistry except the ones developed by MCOs within their networks. Without insurers' mandates to submit specific diagnostic information, and due to the time constraints of busy dental practices, this information is regularly absent from dental charts. This omission considerably limits the value of any retrospective clinical information and makes risk adjustment and outcome assessment very difficult. While an ideal P4P program would reward clinical outcomes, the great majority of medical P4P programs now reward processes of care that are thought to lead to better outcomes. Any dental P4P programs will likely make similar concessions and focus on process until outcomes assessments can be made more feasible. Thus, a P4P program currently could only measure performance through structure, process, patient satisfaction, or attainment of financial goals. Use of clinical outcome measures will only be possible if additional data were submitted by dentists.

#### Electronic dental records

Other important issues in developing outcome indicators are the availability of valid, reliable treatment information and the cost of data collection. Data is valuable as long as it accurately measure clinical reality i.e. actual oral health status, risk factors, diagnoses, treatment, and side effects. Missing data in patient records makes the calculation of performance measures based on dental records difficult [79]. Currently, many dental offices use paper records and collect electronic data only for reimbursement purposes. Assessing performance based on electronic claims information would therefore provide an incomplete picture of clinic activity and thereby limit quality of care information [80]. It may be possible to assess at a practice level the proportion of patients who receive dental sealants, but the diagnostic information on the type of caries and appropriateness of treatment for a particular patient would not be available in claims data. Modifications of the claims codes could address this issue to some extent, but such modifications are not easy to implement and uptake may be variable if reimbursement is not directly tied to the new codes. Absent robust administrative data systems, other performance evaluation methods have been tested [79]. While many MCOs justify the cost of medical records abstraction required to participate in the voluntary Healthcare Data Information Set (HEDIS) public reporting program, the high costs of data abstraction would be a barrier to implementation for individual practices. It will likely be several years before a Universal Electronic Medical Record is adopted and even longer for its dental equivalent. In the meantime, dental P4P program designers must weigh the imperfections of claims data against the feasibility of primary data collection modeled on HEDIS.

In conclusion, dentistry is not ready to follow primary care in implementing value-based purchasing programs. The key elements of a P4P program in general medicine have been identified and can be operationalized: clear objectives, definable units of assessment, valid performance indicators, analysis and interpretation of performance data, performance standards and financial rewards [19]. These elements have not been developed in dentistry yet. Future research is needed to address these issues and to demonstrate a link between financial rewards and performance improvement for dental care.

## Summary

Dentistry may follow medicine by implementing P4P programs to improve dental care quality. P4P may take longer to penetrate dentistry than medicine, due to differences between medical and dental practice, insurance coverage, the dearth of evidence-based guidelines for common dental interventions, and a resultant paucity of evidence-based performance measures. Broad adoption of P4P programs will require the dental profession and health services researchers to: 1) expand the evidence base; 2) create evidence-based clinical guidelines; and 3) create evidence-based performance measures tied to the existing clinical practice guidelines. P4P in dentistry would be a major policy change for the dental profession and the public alike. Based on our review of the literature on evidence-based dentistry and US dental practice patterns, implementing P4P for dental care at this point appears to be premature.

## Competing interests

The authors declare that they have no competing interests.

## Authors' contributions

AVG conceived and drafted the manuscript. JF, BR, AB, GG and MS participated in manuscript design and writing. All authors read and approved the final manuscript.

## Pre-publication history

The pre-publication history for this paper can be accessed here:

http://www.biomedcentral.com/1472-6831/10/9/prepub
